# How Many Days Do We Need? Defining Reliable ActivPAL Monitoring Protocols for Distinct Movement Behaviors in Office Workers

**DOI:** 10.1111/sms.70344

**Published:** 2026-07-11

**Authors:** Ander Espin, Pedro B. Júdice

**Affiliations:** ^1^ Ageing on Research Group University of the Basque Country (UPV/EHU) Leioa Spain; ^2^ Clinical Nursing and Community Health Research Group Biobizkaia Health Research Institute Barakaldo Spain; ^3^ Centro de Investigação Em Educação Física, Desporto, Exercício e Saúde (CIDEFES) Universidade Lusófona Lisbon Portugal; ^4^ Centro de Investigação Formação Inovação E Intervenção Em Desporto (CIFI2D) Universidade Do Porto Porto Portugal

**Keywords:** accelerometry, physical activity, reliability and validity, sedentary behavior

## Abstract

Although device‐measured data provide more reliable estimates of movement behaviors than self‐report questionnaires, device‐monitoring protocols are often demanding in terms of time, logistics, and costs. Identifying the minimum monitoring duration to obtain reliable data is therefore essential, yet evidence for ActivPAL devices remains limited. The objective of this study was to determine the minimum number of days required to reliably estimate movement behaviors using ActivPAL. As a secondary aim, we examined how different combinations of weekdays and weekend days influence reliability. A total of 106 monitoring periods of six consecutive days from adult office workers were analyzed, corresponding to 636 monitored days or 15 264 h collected. Taking the 6‐day period as reference, the reliability of shorter monitoring durations (1–5 days) was assessed. Relative and absolute reliability were confirmed when an intraclass correlation coefficient ≥ 0.85 and a coefficient of variation < 10% were achieved, respectively. Movement behaviors assessed included stepping time, standing time, sedentary time, sitting time, primary lying time, step count, and sit‐to‐stand transitions. Based on these criteria, two monitoring days were sufficient to achieve both relative and absolute reliability for sit‐to‐stand transitions, while 3 days were sufficient for sedentary time, sitting time, and primary lying time. Four days were required to achieve comparable reliability for stepping time, standing time, and step count. Overall, monitoring periods that combined weekdays and weekend days tended to demonstrate higher reliability than those including only weekdays or weekend days. These findings may guide optimization of ActivPAL monitoring protocols, reducing participant and researcher burden while maintaining reliable movement behavior estimates.

## 1 Introduction

Movement behavior is an umbrella term encompassing the full spectrum of an individual's 24‐h movement patterns, including physical activity, sedentary behavior, and sleep [1]. These behaviors are well established as key determinants of health. Extensive evidence indicates that insufficient physical activity, prolonged sedentary behavior, and inadequate sleep are associated with a range of adverse health outcomes, such as premature mortality, cardiometabolic disorders, and certain types of cancer [2, 3, 4]. Consequently, the accurate measurement of movement behaviors is essential, as it enables the identification of individual and population needs, the design of targeted interventions, and the evaluation of their effectiveness.

Traditionally, movement behaviors have been assessed using subjective methods, most commonly self‐report questionnaires such as the International Physical Activity Questionnaire (IPAQ) [5]. Although these instruments are inexpensive, easy to administer, and allow for rapid collection of retrospective data in large populations, they are prone to several sources of bias. Because they rely on participants' self‐reports, these measures are susceptible to recall errors and social desirability bias, often leading individuals to overestimate their actual levels of physical activity and underestimate time spent in sedentary behaviors [6, 7].

Current international physical activity guidelines have largely been informed by evidence derived from self‐reported data [8]. However, recent work using device‐based measures has begun to challenge some of the assumptions that have shaped these recommendations. For example, a recent study using data collected by wearable devices questioned the commonly accepted 1:2 ratio of vigorous: moderate physical activity recommended in current guidelines, indicating that substantially more moderate‐intensity activity may be required to achieve benefits comparable to those of vigorous activity [9]. Such findings expose a clear mismatch between self‐reported and device‐measured estimates and highlight the importance of integrating more objective assessments in research on movement behaviors.

To obtain more objective estimates of movement behaviors, the most common approach to date has been the use of wearable accelerometers, with the ActiGraph device being among the most widely employed in research. ActiGraph is a hip‐ or wrist‐worn triaxial accelerometer that records raw acceleration signals, which are subsequently processed into metrics used to classify physical activity across different intensities [10]. Although it is considered suitable for capturing activity volume and intensity, ActiGraph is less accurate for determining posture, particularly distinguishing between sitting, standing, and lying. For this purpose, the ActivPAL, a thigh‐worn accelerometer and inclinometer that identifies posture based on sensor orientation, is regarded as the gold standard [11, 12]. Its ability to accurately quantify sedentary time, upright time, postural transitions, and stepping activity makes it especially valuable for studies focusing on sedentary behavior and daily movement patterns.

Although accelerometer‐based devices provide more objective and reliable estimates of movement behaviors than self‐report methods, their use can be more demanding in terms of time, logistics, and cost. Researchers must fit participants with the device, ensure it is worn correctly over several days, and then collect it once monitoring is complete. These steps can be challenging for both study teams and participants. As a result, researchers have devoted considerable attention to identifying the minimum wear time needed to obtain reliable data from these devices. Several investigations have done so using ActiGraph monitors. Airlie et al. [13], for example, found that when taking seven consecutive days as the reference, 4 days of monitoring were sufficient to reliably estimate counts per day, counts per minute, physical activity time, and sedentary time. Likewise, Sasaki et al. [14], using a similar 7‐day criterion, reported that between 2.5 and 4.9 days were enough to produce reliable estimates across a range of physical activity and sedentary behavior metrics.

This evidence has important implications for optimizing research efficiency, as it indicates the minimum monitoring required to obtain reliable estimates. However, comparable analyses for ActivPAL remain limited, particularly in office worker populations. Accordingly, the aim of the present study was to determine the minimum number of days necessary to reliably estimate movement behaviors using ActivPAL in office workers, with reliability encompassing both relative and absolute measures. Movement behaviors included stepping time, standing time, sedentary time, sitting time, primary lying time, step count, and sit‐to‐stand transitions. As a secondary aim, we examined how different combinations of weekdays and weekend days influence reliability. To this end, we analyzed ActivPAL data collected in a previous randomized controlled trial [15].

## 2 Materials and Methods

### 2.1 Study Design

This was a reliability study done with data from the SUFHA (acronym for “Stand up for healthy ageing”) randomized controlled trial, which assessed the effects of a sit‐stand desk intervention on movement behaviors in office workers [15]. The present reliability study was conducted in accordance with the Guidelines for Reporting Reliability and Agreement Studies (GRRAS) [16]. The study was approved by the Ethics Committee from the Lusófona University on December 5, 2022 (reference number: D0522), and all participants signed an informed written consent prior to enrollment.

### 2.2 Participants

Data from all participants in the intervention and control groups participating in the SUFHA trial were used in the present study. Participants were all office‐based workers, 20 years old or higher, working at least 60% of full‐time, and spending at least 70% of working time in desk‐related activities. All participants worked on‐site at the university on a Monday‐to‐Friday schedule.

Demographic characteristics were collected through a self‐reported online questionnaire administered at baseline only, as described previously in the SUFHA study protocol [15]. Body weight and height were measured and reported in kilograms (kg) and meters (m), respectively, and body mass index was calculated as body weight (kg)/height (m)^2^. Body composition was assessed using bioelectrical impedance analysis (101 Anniversary, Akern, Florence, Italy), as described in the study protocol [15]. Although body mass index and body composition assessments were repeated during follow‐up, only baseline values were used in the present study to describe participant characteristics.

### 2.3 ActivPAL Measurements

The SUFHA trial included assessments at baseline and at 3, 6, and 9‐month follow‐ups [15]. In this study, valid ActivPAL measurements from all assessment periods in the SUFHA trial were used. To be considered valid for the purpose of the present study, the ActivPAL measurements should contain 24 h monitoring data for six consecutive days. Any measurement containing any non‐wear time (i.e., period during which the device was not worn) during any day was excluded from the analyses, to ensure the validity of the data used. This stringent criterion was adopted to minimize measurement error associated with device non‐wear and to ensure high internal validity for the reliability analyses, as incomplete daily recordings may introduce additional variability unrelated to true movement behaviors. The ActivPAL4 device (PAL Technologies Ltd., Glasgow, UK) was used, and the different movement behaviors were distinguished with the CREA (v1.3) classification algorithm. The ActivPAL device was placed at the participant's right thigh's midpoint with the aid of a waterproof pharmacological dressing. Participants were asked not to ever take off the device, and to maintain their normal routine, while avoiding any aquatic activity that maintained prolonged contact with water (e.g., swimming). In this study, we analyzed the main movement behaviors as provided by ActivPAL: (1) stepping time, (2) standing time, (3) sedentary time (calculated as the sum of sitting time, seated transport time, and secondary lying time), (4) sitting time, and (5) primary lying time, expressed as daily minutes in each of these behaviors, as well as (6) step count and (7) sit to stand transitions, expressed as daily number of steps or transitions, respectively.

### 2.4 Statistical Analyses

Descriptive statistics were based on the mean and the standard deviation for continuous variables and frequency counts and percentages for categorical variables. For the reliability analyses, the mean of the six consecutive days of data collection was taken as the valid reference for each movement behavior. Considering the 6‐day reference, the reliability of different monitoring durations was assessed: i.e., first single day of monitoring versus 6‐day reference, mean of first 2 days versus 6‐day reference, mean of first 3 days versus 6‐day reference, mean of first 4 days versus 6‐day reference, and mean of first 5 days versus six‐day reference. To ensure a comprehensive reliability analysis, both relative and absolute reliability measures were used. For the relative reliability, the intraclass correlation coefficient (ICC) and its 95% confidence interval (CI) was used, applying a two‐way mixed effects, single measurement, absolute agreement model [17]. Regarding the interpretation of the ICC, while the identified studies similar to ours established a threshold of ≥ 0.8 [14, 18, 19], we opted for considering a more strict ICC value of 0.85 or higher to confirm relative reliability, in order to get closer to the ≥ 0.9 criterion which has been usually regarded as excellent reliability [17]. For the absolute reliability, the standard error of measurement (SEM) was used, calculated as the square root of the mean square error term in a repeated measures ANOVA [20]. Derived from the SEM, the coefficient of variation (CV) was calculated, by obtaining the percentage of SEM relative to the 6‐day reference in each analysis. Regarding the interpretation of the CV, in the absence of a universally accepted criterion, a commonly used threshold of < 10% was established to confirm absolute reliability [20]. CV values were calculated using unrounded data, and classification relative to the 10% threshold was based on these exact values. For reporting purposes, CVs are presented to one decimal place. Although the main reliability analyses were performed without taking into account if the different ActivPAL monitoring periods included weekdays or weekend days, we carried out a secondary analysis to assess the influence of monitoring different combinations of weekday/weekend days. This was possible because the first monitoring day (Monday to Saturday) varied among participants. In this secondary analysis, the statistical procedures and their interpretation criteria were the same as described above for the main analysis. The only difference was that, taking the 6‐day monitoring period as a reference again, all possible combinations regarding weekday/weekend days were compared in terms of reliability: i.e., first single‐day measurements were analyzed separately as first single weekday or first single weekend day; mean of first 2 days measurements were analyzed separately as mean of first two weekdays, one weekday plus one weekend day, or two weekend days; and the same logic was followed for 3, 4 and 5‐day monitoring periods. Given that multiple monitoring periods were obtained from the same participants, the dataset includes repeated measures. Each monitoring period was treated as an independent observation, consistent with previous studies, although this does not fully account for within‐subject correlation and may affect the precision of the estimates. All statistical analyses were performed using IBM SPSS Statistics package for Windows version 31.0 (IBM Corp., Armonk, NY, USA).

## 3 Results

### 3.1 Participants

Valid ActivPAL data from all the participants in the SUFHA trial (*n* = 38) were available for the purpose of the present study. Participants had been equally allocated to the intervention (*n* = 19) and control (*n* = 19) groups at baseline. The characteristics of the participants are reported in Table 1. The mean age was 43.8 years, and the majority were women (76.3%) and had Portuguese nationality (89.5%).

**TABLE 1:** Descriptive data of the study participants (*N* = 38).

Variable	Value
Age (years)	43.8 ± 8.0
Gender	
Woman	29 (76.3%)
Man	9 (23.7%)
Nationality	
Portuguese	34 (89.5%)
Brazilian	1 (2.6%)
Dual (Portuguese + any other)	3 (7.8%)
Education	
High school	11 (28.9%)
Bachelor's degree	18 (47.4%)
Master's degree	5 (13.2%)
PhD degree	4 (10.5%)
Occupation	
Academic/administrative services	15 (39.5%)
Management/administrative services	7 (18.4%)
Financial services	3 (7.9%)
Other	13 (34.2%)
Years of service	14.9 ± 10.7
Daily working time (minutes/day)	474.0 ± 78.8
Smoking status	
Current	5 (13.2%)
Former	12 (31.6%)
Occasionally	3 (7.9%)
Never	18 (47.4%)
Chronic disease	
Yes	8 (21.1%)
No	30 (78.9%)
Body weight (kg)	77.0 ± 19.4
Height (m)	1.63 ± 0.10
Body mass index (kg/m^2^)	28.8 ± 6.9
Body fat (%)	37.4 ± 7.8

*Note:* Data are mean ± standard deviation or frequency (%).

### 3.2 Valid ActivPAL Measurements

A total of 106 valid ActivPAL measurements were available for the analyses, corresponding to a total of 636 days or 15 264 h monitored. This number is lower than the theoretical maximum due to missing assessments across study waves and exclusion of recordings not meeting validity criteria (e.g., non‐wear time or incomplete six consecutive days of 24‐h monitoring). Participants contributed between one and four valid monitoring periods. Regarding seasonality, 60 monitoring periods (57%) were collected during autumn‐winter and 46 (43%) during spring–summer. The first monitoring day of these measurements was Monday in two cases (2%), Tuesday in 14 cases (13%), Wednesday in 20 cases (19%), Thursday in 29 cases (27%), Friday in 19 cases (18%), and Saturday in 22 cases (21%).

### 3.3 Main Reliability Analysis

The reliability of different ActivPAL monitoring durations for each movement behavior can be observed in Table 2.

**TABLE 2:** Comparison of ActivPAL‐derived movement behaviors across different monitoring durations relative to the 6‐day reference period.

Movement behavior	Mean (SD)	ICC (95% CI)	SEM (CV)
Stepping time, minutes			
1 day	97.0 (32.7)	0.63 (0.50–0.73)	18.2 (18.8%)
2 days	95.6 (27.1)	0.79 (0.71–0.85)	12.3 (12.9%)
3 days	94.7 (25.6)	0.86 (0.80–0.90)	9.9 (10.5%)
4 days	94.6 (26.2)	0.93 (0.90–0.95)	7.0 (7.4%)
5 days	95.3 (27.1)	0.98 (0.96–0.98)	4.3 (4.5%)
6 days (reference)	94.8 (26.5)		
Standing time, minutes			
1 day	282 (132)	0.76 (0.66–0.83)	58 (20.6%)
2 days	279 (118)	0.85 (0.79–0.90)	42 (15.1%)
3 days	276 (109)	0.93 (0.90–0.95)	28 (10.1%)
4 days	274 (107)	0.96 (0.95–0.97)	20 (7.3%)
5 days	273 (103)	0.99 (0.98–0.99)	11 (4.0%)
6 days (reference)	272 (100)		
Sedentary time, minutes			
1 day	561 (141)	0.66 (0.54–0.76)	72 (12.8%)
2 days	562 (125)	0.78 (0.69–0.84)	55 (9.8%)
3 days	557 (118)	0.86 (0.80–0.90)	42 (7.5%)
4 days	555 (116)	0.93 (0.90–0.95)	29 (5.2%)
5 days	560 (111)	0.97 (0.96–0.98)	17 (3.0%)
6 days (reference)	561 (106)		
Sitting time, minutes			
1 day	473 (140)	0.71 (0.60–0.79)	67 (14.2%)
2 days	462 (130)	0.79 (0.70–0.85)	55 (11.9%)
3 days	457 (123)	0.87 (0.82–0.91)	41 (9.0%)
4 days	454 (116)	0.93 (0.90–0.95)	29 (6.4%)
5 days	455 (110)	0.98 (0.96–0.98)	17 (3.7%)
6 days (reference)	455 (107)		
Primary lying time, minutes			
1 day	499 (92)	0.52 (0.37–0.65)	57 (11.4%)
2 days	503 (78)	0.75 (0.66–0.83)	37 (7.4%)
3 days	511 (75)	0.87 (0.82–0.91)	26 (5.1%)
4 days	515 (74)	0.91 (0.88–0.94)	21 (4.1%)
5 days	511 (72)	0.97 (0.95–0.98)	13 (2.5%)
6 days (reference)	509 (73)		
Step count, number			
1 day	7995 (3002)	0.65 (0.52–0.75)	1603 (20.1%)
2 days	7761 (2508)	0.82 (0.74–0.87)	1055 (13.6%)
3 days	7634 (2348)	0.88 (0.83–0.92)	819 (10.7%)
4 days	7598 (2386)	0.94 (0.91–0.96)	594 (7.8%)
5 days	7681 (2443)	0.97 (0.96–0.98)	391 (5.1%)
6 days (reference)	7653 (2394)		
Sit to stand transitions, number			
1 day	46.2 (15.0)	0.72 (0.62–0.80)	6.8 (14.7%)
2 days	46.8 (13.4)	0.85 (0.79–0.90)	4.5 (9.6%)
3 days	46.4 (11.9)	0.93 (0.90–0.95)	3.0 (6.5%)
4 days	45.6 (11.2)	0.95 (0.93–0.97)	2.4 (5.3%)
5 days	45.5 (10.5)	0.99 (0.98–0.99)	1.2 (2.6%)
6 days (reference)	45.6 (10.4)		

Abbreviations: CI, confidence interval; CV, coefficient of variation; ICC, intraclass correlation coefficient; SD, standard deviation; SEM, standard error of measurement.

For stepping time, the average was 94.8 daily stepping minutes during the 6‐day reference period. Monitoring durations of 3 days and above provided ≥ 0.85 ICC values (e.g., ICC = 0.86 for 3 days), and monitoring durations of 4 days and above provided < 10% CV percentages (e.g., CV = 7.4% for 4 days).

For standing time, the average was 272 daily standing minutes during the 6‐day reference period. Monitoring durations of 2 days and above provided ≥ 0.85 ICC values (e.g., ICC = 0.85 for 2 days), and monitoring durations of 4 days and above provided < 10% CV percentages (e.g., CV = 7.3% for 4 days).

For sedentary time, the average was 561 daily sedentary minutes during the 6‐day reference period. Monitoring durations of 3 days and above provided ≥ 0.85 ICC values (e.g., ICC = 0.86 for 3 days), and monitoring durations of 2 days and above provided < 10% CV percentages (e.g., CV = 9.8% for 2 days).

For sitting time, the average was 455 daily sitting minutes during the 6‐day reference period. Monitoring durations of 3 days and above provided ≥ 0.85 ICC values and < 10% CV percentages (e.g., ICC = 0.87 and CV = 9.0% for 3 days).

For primary lying time, the average was 509 daily lying minutes during the 6‐day reference period. Monitoring durations of 3 days and above provided ≥ 0.85 ICC values (e.g., ICC = 0.87 for 3 days), and monitoring durations of 2 days and above provided < 10% CV percentages (e.g., CV = 7.4% for 2 days).

For the step count, the average was 7653 daily steps during the 6‐day reference period. Monitoring durations of 3 days and above provided ≥ 0.85 ICC values (e.g., ICC = 0.88 for 3 days), and monitoring durations of 4 days and above provided < 10% CV percentages (e.g., CV = 7.8% for 4 days).

For the sit to stand transitions, the average was 45.6 daily transitions during the 6‐day reference period. Monitoring durations of 2 days and above provided ≥ 0.85 ICC values and < 10% CV percentages (e.g., ICC = 0.85 and CV = 9.6% for 2 days).

**FIGURE 1:**
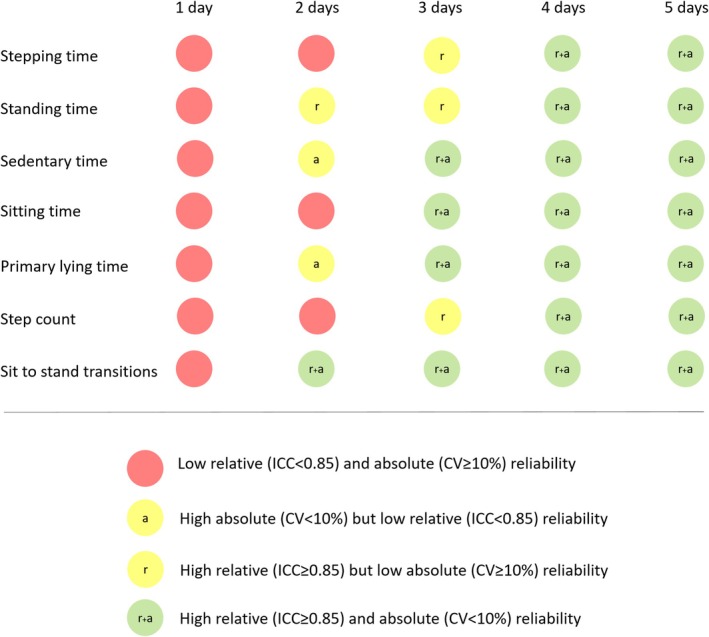
Graphical summary of the reliability of different monitoring durations relative to the 6‐day reference period using ActivPAL.

Figure 1 depicts a graphical summary of the reliability of different monitoring durations. Reliability outcomes are represented using a combined color‐ and letter‐coded system to facilitate interpretation. Specifically, absence of a letter indicates low relative and absolute reliability (red); the letter “a” denotes high absolute but low relative reliability (yellow); the letter “r” denotes high relative but low absolute reliability (yellow); and the combination “*r* + a” denotes high relative and absolute reliability (green). In general, to get both relative and absolute reliabilities according to the criteria established in the present study, 2 days of monitoring were sufficient for sit‐to‐stand transitions, while 3 days were sufficient for sedentary time, sitting time, and primary lying time. On the other hand, 4 days would be necessary to obtain such reliabilities for stepping time, standing time, and the step count.

### 3.4 Influence of Different Combinations of Weekdays and Weekend Days

The secondary analyses with the reliability of diverse weekday/weekend day combinations are reported in Table S1. In general, it can be observed that the minimum number of monitoring days to get reliable estimates remains similar to the main analysis. Importantly, monitoring periods which combine weekday and weekend days, as opposed to those comprising only weekdays or weekend days in isolation, seem to provide higher reliability. These findings should be interpreted with caution given the reduced sample size for specific weekday/weekend combinations and should be considered exploratory in nature.

## 4 Discussion

The present study sought to determine the minimum number of monitoring days required to obtain reliable estimates of distinct movement behaviors using the ActivPAL4 device. Considering an ICC of ≥ 0.85 and a CV of < 10%, and taking a 6‐day period as the reference, 2 days of monitoring were sufficient to reliably characterize sit‐to‐stand transitions, while 3 days were sufficient for sedentary time, sitting time, and primary lying time, and 4 days were necessary for stepping time, standing time, and step count. These findings suggest that fewer monitoring days may be adequate for behaviors at the lower end of the physical activity spectrum, whereas activities higher in the spectrum might require slightly longer monitoring periods to achieve similar reliability.

Regarding our secondary aim, the analyses examining different combinations of weekdays and weekend days suggested that mixed monitoring periods generally tended to yield higher reliability than periods composed exclusively of weekdays or exclusively of weekend days. In general, combinations containing only weekdays or a higher proportion of weekdays tended to underestimate overall physical activity relative to the 6‐day reference period, whereas combinations composed solely or predominantly of weekend days tended to overestimate it. This discrepancy could be likely attributable to characteristic behavioral patterns of office workers, with more sedentary routines during the workweek and comparatively higher levels of activity during weekends [21]. These findings suggest that, when monitoring periods shorter than a full week are used with ActivPAL, incorporating both weekday and weekend days may enhance the representativeness of the data. This aligns with previous ActivPAL‐based research indicating that combining weekday and weekend monitoring yields more precise estimates of movement behaviors [18]. However, given that our study was not specifically designed to address this question and that certain weekday/weekend combinations were based on limited observations, future research specifically designed to evaluate optimal day composition for ActivPAL monitoring is warranted.

The implications of these findings are highly relevant for researchers planning studies involving ActivPAL‐based assessment of movement behaviors. Considering that reliable estimates can be achieved with 2–4 days of monitoring, several operational advantages could be expected. Shorter monitoring periods facilitate faster data collection, reduce logistical demands, and optimize the use of devices. Moreover, reducing the required wear time lessens participant burden, which may improve adherence to monitoring protocols and decrease the likelihood of data loss due to device non‐compliance. This is particularly relevant given that previous research reported that most participants found continuous 7‐day ActivPAL monitoring too burdensome, with many experiencing adherence problems and over half indicating they would be unwilling to wear the device again [22]. Together, the shorter monitoring periods and reduced participant burden could collectively translate into lower study costs and more streamlined research workflows. Nevertheless, it remains essential that future studies employing ActivPAL explicitly report the duration and composition of their monitoring protocols, as these methodological choices could directly influence the interpretation, comparability, and reproducibility of the findings.

When considering our findings in the context of the existing literature, it becomes evident that relatively few studies have examined the reliability of ActivPAL‐derived movement behaviors across multiple monitoring durations, and those that have are largely focused on highly specific populations. Prior investigations have included older adults living in residential aged‐care facilities [19, 23], healthy middle‐aged and older women participating in a mammographic breast density study [24], adolescent females [25], and patients undergoing hemodialysis [26]. As such, their applicability to community‐dwelling adult populations is limited. To the best of our knowledge, the only study more comparable to ours is that by Aguilar‐Farias et al. [18], conducted among Chilean working adults. Similar to our results, and taking a 7‐day period as reference, Aguilar‐Farias and colleagues reported that 3 days of monitoring were sufficient to obtain reliable estimates of sit to stand transitions. For sitting/lying, standing, and stepping time, they found that a slightly longer monitoring period of 5 days was required. Evidence from other populations using ActivPAL devices and 7‐day reference periods has also reported that approximately 3–4 days of monitoring are sufficient to obtain reliable estimates of movement behaviors [19, 24], further supporting the general pattern observed in the present study. Consistent with our secondary analyses, Aguilar‐Farias and colleagues also concluded that estimates were more precise when at least one weekend day was included in the monitoring protocol.

Despite these similarities, several methodological and conceptual differences distinguish our study from that of Aguilar‐Farias and colleagues. To begin with, we examined a wider set of movement behaviors. Beyond the outcomes reported in the Chilean study, we distinguished sedentary time and sitting time, and we also incorporated step count and primary lying time. This distinction is important because such movement behaviors are increasingly recognized as key indicators in public health research [27, 28, 29], and optimizing their assessment is therefore essential. There were also differences in data inclusion criteria that can strongly impact the results. Aguilar‐Farias and colleagues accepted days with at least 10 h of valid wear time, while we required complete 24‐h monitoring days. Our dataset also included slightly more valid measurements, with 106 cases compared to their 90. Finally, the approach used to construct different monitoring durations differed between studies. In our study, 1‐day, 2‐day, and multi‐day periods were based on the first consecutive days of monitoring, reflecting the sequence that would occur in real‐world data collection. In contrast, Aguilar‐Farias et al. selected days at random for each monitoring duration. While this approach may better capture variability across different days of the week, it may be less reflective of how monitoring is typically conducted in applied settings, where devices are worn over consecutive days. In this sense, our approach aimed to provide reliability estimates that are more directly aligned with real‐world data collection protocols, while also capturing potential temporal effects such as behavioral reactivity or adaptation over the monitoring period. However, we acknowledge that using only the first consecutive days may introduce temporal order effects, as behavior during initial wear days may differ from later days.

Several strengths of the present study should be highlighted. First, we assessed both relative and absolute reliability, providing a comprehensive evaluation. We applied more stringent criteria than previous studies [14, 18, 19], using an ICC threshold of 0.85 rather than 0.80 to define acceptable relative reliability. Second, according to the guidelines by Koo and Li [17], reliability studies generally require at least 30 cases as a rule of thumb, and our study included 106 valid measurement cases. Third, the first day of monitoring was quite evenly distributed across the days of the week, from Monday to Saturday, reducing potential bias related to day‐specific activity patterns. Fourth, by including measurements from multiple follow‐up periods within the trial, our study captures a degree of seasonal variation in movement behaviors, addressing a limitation noted in prior research [18]. Finally, we provide a graphical summary of the reliability results, offering a clear and practical tool for future researchers. This visual overview allows investigators to make evidence‐based decisions regarding the number of monitoring days required for specific movement behaviors when designing studies using ActivPAL devices, as not all behaviors may be the target in specific investigations.

However, several limitations of the present study should also be acknowledged. First, the sample size for comparisons involving different combinations of weekday and weekend days was relatively limited, which may reduce the precision of estimates for some combinations. Second, although 106 monitoring periods were analyzed, these were derived from 38 participants, resulting in repeated measurements within individuals. As observations were treated as independent, within‐subject correlation may have led to an underestimation of variability. In addition, the modest number of participants may limit the generalizability of the findings and the stability of estimates across different populations. Therefore, results should be interpreted with some caution, and future studies could consider using approaches that explicitly account for within‐subject clustering (e.g., multilevel models). In particular, the study population consisted exclusively of Portuguese office workers with specific demographic and occupational characteristics, which may further limit the generalizability to other populations with different movement patterns. The predominance of women in the sample may also affect the applicability of the results to more gender‐balanced populations. Moreover, the degree of day‐to‐day variability in movement behaviors may differ across populations, which could influence reliability estimates and the minimum number of monitoring days required. For example, more structured occupational settings may show lower within‐person variability, whereas populations with more heterogeneous or less predictable daily routines may require longer monitoring periods to achieve comparable reliability. Therefore, the present findings should be interpreted within the context of an office‐working adult population. Third, although we applied established and relatively stringent criteria to define acceptable reliability, using alternative thresholds (e.g., different ICC and CV cut‐offs) could lead to slightly different interpretations of required monitoring durations. Fourth, the use of a 6‐day reference period instead of the more commonly adopted seven‐day monitoring protocol may be considered a limitation. This decision was driven by the application of strict data inclusion criteria requiring complete 24‐h recordings with no non‐wear time, which would have substantially reduced the sample size and statistical power if extended to 7 days. Although each monitoring period spanned six consecutive days and therefore included both weekday and weekend days, and variability across the week was further preserved at the group level due to different starting days among participants, this methodological choice may limit direct comparability with previous studies using 7‐day reference periods. Fifth, the use of strict data inclusion criteria (i.e., complete 24‐h monitoring with no non‐wear time) may limit generalizability to studies using less stringent protocols, although this approach was chosen to minimize measurement error and improve the precision of reliability estimates.

Future research could build on the present findings in several ways. Although our study included some degree of seasonal variation, studies specifically designed to assess potential differences in reliability across seasons or environmental conditions, such as winter versus summer or wet versus dry periods, could provide valuable insights, given that physical activity patterns can vary throughout the year [30]. In addition, studies specifically aimed at examining the influence of weekday and weekend day combinations on reliability would be beneficial. Such studies could ensure sufficiently large and evenly distributed sample sizes across all possible day combinations, allowing for more precise estimates and a clearer understanding of how day composition affects the reliability of ActivPAL‐derived movement behaviors. These approaches would further refine evidence‐based guidelines for monitoring protocols and enhance the applicability of findings across diverse populations and settings.

## 5 Perspective

Future research should extend these findings by testing ActivPAL monitoring protocols across more diverse populations, including different age groups, occupational settings, and clinical conditions, where movement behavior patterns and variability may differ substantially. Ensuring more balanced samples in terms of sex and other key characteristics would further strengthen the applicability of resulting recommendations. Further work is also needed to better understand how the composition of monitoring days influences reliability. While combining weekdays and weekend days appears advantageous, studies specifically designed with sufficiently large and evenly distributed samples across all possible day combinations are required to provide more precise and definitive guidance. In addition, contextual factors such as seasonal variation and environmental conditions should be more explicitly incorporated into study designs, as these may influence day‐to‐day movement variability. Finally, future studies should examine how different reliability thresholds and analytical approaches, such as alternative ICC criteria or reference periods, influence the estimated minimum number of monitoring days. Advancing these areas will contribute to more robust, generalizable, and context‐sensitive ActivPAL monitoring protocols.

## Funding

This work was supported by Instituto Lusófono de Investigação e Desenvolvimento (ILIND) “Fazer +”, FAZER+/ILIND/CIDEFES/1/2022 and Ministerio de Ciencia, Innovación y Universidades, CAS24/00318.

## Conflicts of Interest

The authors declare no conflicts of interest.

## Supporting information


**Table S1:** Comparison of ActivPAL‐derived movement behaviors across different combinations of monitoring days relative to the 6‐day reference period.

## Data Availability

The data that support the findings of this study are available from the corresponding author upon reasonable request.
