# Supplementary material for: How Many Days Do We Need? Defining Reliable ActivPAL Monitoring Protocols for Distinct Movement Behaviors in Office Workers
Source: Scand J Med Sci Sports. 2026 Jul 11;36(7):e70344. doi: 10.1111/sms.70344 (PMC13355939; doi:10.1111/sms.70344)
Supplement: Supplementary file 1 — Table S1: Comparison of ActivPAL‐derived movement behaviors across different combinations of monitoring days relative to the 6‐day reference period. [file SMS-36-e70344-s001.docx]

**Supplementary Table 1** Comparison of ActivPAL-derived movement behaviors across different combinations of monitoring days relative to the 6-day reference period

| Monitoring days | n | Mean (SD) | Mean (SD) 6 days (ref) | ICC (95% CI) | SEM (CV) |
| --- | --- | --- | --- | --- | --- |
| Stepping time (min) |  |  |  |  |  |
| 1 day (1w) | 84 | 94.9 (30.6) | 95.5 (27.8) | 0.71 (0.59-0.80) | 15.8 (17%) |
| 1 day (1e) | 22 | 105.0 (39.4) | 91.8 (20.9) | 0.39 (0.00-0.69) | 24.1 (26%) |
| 2 days (2w) | 65 | 93.8 (25.9) | 94.7 (27.0) | 0.78 (0.66-0.86) | 12.5 (13%) |
| 2 days (1w+1e) | 19 | 101.2 (30.9) | 98.5 (31.1) | 0.87 (0.71-0.95) | 11.1 (11%) |
| 2 days (2e) | 22 | 96.1 (27.8) | 91.8 (20.9) | 0.74 (0.47-0.88) | 12.6 (14%) |
| 3 days (3w) | 36 | 90.0 (24.2) | 94.9 (27.7) | 0.76 (0.58-0.87) | 12.5 (13%) |
| 3 days (2w+1e) | 29 | 98.5 (26.9) | 94.3 (26.6) | 0.92 (0.83-0.96) | 7.0 (7%) |
| 3 days (1w+2e) | 41 | 96.2 (25.9) | 94.9 (26.0) | 0.90 (0.83-0.95) | 8.2 (9%) |
| 4 days (4w) | 16 | 90.8 (23.7) | 97.2 (24.9) | 0.87 (0.62-0.95) | 7.9 (8%) |
| 4 days (3w+1e) | 20 | 95.8 (29.1) | 93.1 (30.3) | 0.92 (0.80-0.97) | 8.6 (9%) |
| 4 days (2w+2e) | 70 | 95.1 (26.2) | 94.7 (26.1) | 0.95 (0.92-0.97) | 5.8 (6%) |
| 5 days (4w+1e) | 14 | 96.6 (28.9) | 96.5 (26.7) | 0.97 (0.90-0.99) | 5.2 (5%) |
| 5 days (3w+2e) | 90 | 95.2 (27.3) | 94.3 (26.9) | 0.98 (0.97-0.99) | 3.9 (4%) |
| Standing time (min) |  |  |  |  |  |
| 1 day (1w) | 84 | 280 (129) | 279 (102) | 0.81 (0.72-0.87) | 51 (18%) |
| 1 day (1e) | 22 | 290 (148) | 247 (90) | 0.60 (0.25-0.81) | 75 (30%) |
| 2 days (2w) | 65 | 280 (123) | 279 (106) | 0.89 (0.83-0.93) | 38 (13%) |
| 2 days (1w+1e) | 19 | 288 (102) | 280 (89) | 0.87 (0.70-0.95) | 35 (12%) |
| 2 days (2e) | 22 | 266 (119) | 247 (90) | 0.71 (0.42-0.87) | 57 (23%) |
| 3 days (3w) | 36 | 255 (110) | 264 (100) | 0.91 (0.83-0.95) | 32 (12%) |
| 3 days (2w+1e) | 29 | 305 (111) | 297 (112) | 0.97 (0.94-0.99) | 18 (6%) |
| 3 days (1w+2e) | 41 | 275 (105) | 262 (90) | 0.91 (0.83-0.95) | 29 (11%) |
| 4 days (4w) | 16 | 243 (113) | 262 (90) | 0.91 (0.75-0.97) | 29 (11%) |
| 4 days (3w+1e) | 20 | 273 (111) | 267 (110) | 0.96 (0.90-0.98) | 23 (9%) |
| 4 days (2w+2e) | 70 | 281 (104) | 277 (100) | 0.98 (0.96-0.99) | 16 (6%) |
| 5 days (4w+1e) | 14 | 261 (103) | 266 (96) | 0.98 (0.93-0.99) | 16 (6%) |
| 5 days (3w+2e) | 90 | 276 (104) | 274 (102) | 0.99 (0.99-0.99) | 10 (4%) |
| Sedentary time (min) |  |  |  |  |  |
| 1 day (1w) | 84 | 574 (132) | 553 (107) | 0.78 (0.67-0.85) | 56 (10%) |
| 1 day (1e) | 22 | 511 (163) | 591 (101) | 0.40 (0.02-0.69) | 99 (17%) |
| 2 days (2w) | 65 | 580 (123) | 557 (111) | 0.81 (0.69-0.88) | 50 (9%) |
| 2 days (1w+1e) | 19 | 542 (115) | 541 (93) | 0.86 (0.66-0.94) | 41 (8%) |
| 2 days (2e) | 22 | 527 (136) | 591 (101) | 0.68 (0.19-0.87) | 58 (10%) |
| 3 days (3w) | 36 | 604 (119) | 574 (117) | 0.83 (0.66-0.92) | 45 (8%) |
| 3 days (2w+1e) | 29 | 527 (103) | 535 (101) | 0.90 (0.80-0.95) | 32 (6%) |
| 3 days (1w+2e) | 41 | 536 (117) | 568 (99) | 0.86 (0.63-0.94) | 35 (6%) |
| 4 days (4w) | 16 | 624 (122) | 587 (104) | 0.90 (0.42-0.97) | 26 (4%) |
| 4 days (3w+1e) | 20 | 559 (126) | 564 (127) | 0.90 (0.78-0.96) | 40 (7%) |
| 4 days (2w+2e) | 70 | 539 (108) | 554 (101) | 0.95 (0.89-0.98) | 20 (4%) |
| 5 days (4w+1e) | 14 | 582 (108) | 571 (100) | 0.98 (0.92-0.99) | 15 (3%) |
| 5 days (3w+2e) | 90 | 553 (109) | 557 (106) | 0.97 (0.96-0.98) | 17 (3%) |
| Sitting time (min) |  |  |  |  |  |
| 1 day (1w) | 84 | 490 (133) | 451 (111) | 0.79 (0.60-0.88) | 51 (11%) |
| 1 day (1e) | 22 | 407 (151) | 471 (91) | 0.45 (0.07-0.73) | 88 (19%) |
| 2 days (2w) | 65 | 485 (131) | 452 (111) | 0.83 (0.67-0.90) | 47 (10%) |
| 2 days (1w+1e) | 19 | 456 (129) | 447 (116) | 0.85 (0.64-0.94) | 49 (11%) |
| 2 days (2e) | 22 | 399 (111) | 471 (91) | 0.63 (-0.01-0.87) | 47 (10%) |
| 3 days (3w) | 36 | 503 (137) | 460 (120) | 0.87 (0.53-0.95) | 37 (8%) |
| 3 days (2w+1e) | 29 | 436 (103) | 443 (99) | 0.91 (0.82-0.96) | 30 (7%) |
| 3 days (1w+2e) | 41 | 432 (114) | 460 (103) | 0.86 (0.68-0.93) | 37 (8%) |
| 4 days (4w) | 16 | 518 (137) | 468 (117) | 0.89 (0.15-0.97) | 27 (6%) |
| 4 days (3w+1e) | 20 | 458 (132) | 454 (125) | 0.95 (0.87-0.98) | 30 (7%) |
| 4 days (2w+2e) | 70 | 438 (103) | 453 (101) | 0.94 (0.89-0.97) | 22 (5%) |
| 5 days (4w+1e) | 14 | 474 (129) | 453 (117) | 0.97 (0.77-0.99) | 16 (4%) |
| 5 days (3w+2e) | 90 | 448 (106) | 453 (106) | 0.98 (0.97-0.99) | 16 (3%) |
| Primary lying time (min) |  |  |  |  |  |
| 1 day (1w) | 84 | 490 (83) | 509 (69) | 0.67 (0.53-0.78) | 42 (8%) |
| 1 day (1e) | 22 | 534 (116) | 509 (89) | 0.20 (-0.23-0.57) | 92 (18%) |
| 2 days (2w) | 65 | 485 (75) | 505 (74) | 0.75 (0.59-0.85) | 36 (7%) |
| 2 days (1w+1e) | 19 | 508 (47) | 520 (47) | 0.80 (0.55-0.92) | 20 (4%) |
| 2 days (2e) | 22 | 550 (90) | 509 (89) | 0.76 (0.29-0.91) | 36 (7%) |
| 3 days (3w) | 36 | 490 (73) | 499 (73) | 0.85 (0.72-0.92) | 28 (6%) |
| 3 days (2w+1e) | 29 | 509 (82) | 514 (77) | 0.86 (0.72-0.93) | 30 (6%) |
| 3 days (1w+2e) | 41 | 532 (68) | 514 (72) | 0.91 (0.69-0.97) | 17 (3%) |
| 4 days (4w) | 16 | 481 (68) | 493 (73) | 0.88 (0.71-0.96) | 23 (5%) |
| 4 days (3w+1e) | 20 | 511 (73) | 503 (74) | 0.83 (0.63-0.93) | 30 (6%) |
| 4 days (2w+2e) | 70 | 524 (74) | 514 (74) | 0.94 (0.89-0.97) | 16 (3%) |
| 5 days (4w+1e) | 14 | 500 (56) | 506 (66) | 0.92 (0.77-0.97) | 18 (4%) |
| 5 days (3w+2e) | 90 | 515 (73) | 512 (73) | 0.97 (0.96-0.98) | 12 (2%) |
| Step count (n) |  |  |  |  |  |
| 1 day (1w) | 84 | 7957 (2880) | 7690 (2497) | 0.70 (0.57-0.79) | 1487 (19%) |
| 1 day (1e) | 22 | 8137 (3503) | 7513 (1998) | 0.50 (0.12-0.76) | 2014 (27%) |
| 2 days (2w) | 65 | 7885 (2475) | 7631 (2380) | 0.79 (0.68-0.87) | 1103 (14%) |
| 2 days (1w+1e) | 19 | 7863 (2787) | 7890 (2925) | 0.89 (0.75-0.96) | 953 (12%) |
| 2 days (2e) | 22 | 7303 (2420) | 7513 (1998) | 0.80 (0.59-0.91) | 993 (13%) |
| 3 days (3w) | 36 | 7486 (2241) | 7637 (2397) | 0.81 (0.65-0.90) | 1028 (13%) |
| 3 days (2w+1e) | 29 | 8030 (2540) | 7625 (2400) | 0.93 (0.83-0.97) | 628 (8%) |
| 3 days (1w+2e) | 41 | 7483 (2325) | 7687 (2446) | 0.92 (0.85-0.95) | 685 (9%) |
| 4 days (4w) | 16 | 7793 (2262) | 8008 (2181) | 0.90 (0.74-0.96) | 721 (9%) |
| 4 days (3w+1e) | 20 | 7559 (2472) | 7340 (2574) | 0.92 (0.80-0.97) | 735 (10%) |
| 4 days (2w+2e) | 70 | 7565 (2421) | 7661 (2410) | 0.96 (0.93-0.97) | 509 (7%) |
| 5 days (4w+1e) | 14 | 7815 (2376) | 7713 (2177) | 0.96 (0.89-0.99) | 445 (6%) |
| 5 days (3w+2e) | 90 | 7624 (2479) | 7590 (2436) | 0.98 (0.97-0.99) | 375 (5%) |
| Sit to stand transitions (n) |  |  |  |  |  |
| 1 day (1w) | 84 | 46.1 (14.4) | 45.1 (9.9) | 0.74 (0.62-0.82) | 6.3 (14%) |
| 1 day (1e) | 22 | 46.3 (17.6) | 47.5 (12.1) | 0.70 (0.40-0.86) | 8.4 (18%) |
| 2 days (2w) | 65 | 46.4 (12.9) | 44.3 (9.7) | 0.85 (0.75-0.91) | 4.3 (10%) |
| 2 days (1w+1e) | 19 | 48.9 (14.4) | 47.7 (10.3) | 0.90 (0.77-0.96) | 3.9 (8%) |
| 2 days (2e) | 22 | 45.8 (14.5) | 47.5 (12.1) | 0.84 (0.67-0.93) | 5.3 (11%) |
| 3 days (3w) | 36 | 44.6 (11.7) | 42.8 (9.7) | 0.91 (0.82-0.96) | 3.0 (7%) |
| 3 days (2w+1e) | 29 | 46.5 (10.5) | 46.2 (9.6) | 0.94 (0.87-0.97) | 2.5 (5%) |
| 3 days (1w+2e) | 41 | 47.9 (13.0) | 47.6 (11.2) | 0.93 (0.88-0.96) | 3.2 (7%) |
| 4 days (4w) | 16 | 43.5 (9.7) | 42.9 (7.6) | 0.96 (0.93-0.97) | 2.4 (6%) |
| 4 days (3w+1e) | 20 | 44.2 (12.3) | 42.7 (11.3) | 0.95 (0.87-0.98) | 2.4 (6%) |
| 4 days (2w+2e) | 70 | 46.4 (11.2) | 47.0 (10.5) | 0.96 (0.93-0.97) | 2.2 (5%) |
| 5 days (4w+1e) | 14 | 44.8 (7.8) | 44.3 (6.6) | 0.95 (0.85-0.98) | 1.7 (4%) |
| 5 days (3w+2e) | 90 | 45.9 (10.8) | 46.0 (10.8) | 0.99 (0.98-0.99) | 1.2 (3%) |

Note: n refers to the amount of valid ActivPAL measurements; 5 days (5w) is not included due to low number of cases (n=2); CI: confidence interval; CV: coefficient of variation; e: weekend day; ICC: intraclass correlation coefficient; min: minutes; SD: standard deviation; SEM: standard error of measurement; w: weekday
